# Special Issue “Human Betaretrovirus and Related Diseases”

**DOI:** 10.3390/v14122792

**Published:** 2022-12-15

**Authors:** Andrew L. Mason

**Affiliations:** Center of Excellence for Gastrointestinal Inflammation and Immunity Research, University of Alberta, Edmonton, AB T6G 2E1, Canada; andrew.mason@ualberta.ca

A betaretrovirus resembling mouse mammary tumor virus (MMTV) was first linked with human breast cancer over 50 years ago. It was originally referred to as human mammary tumor virus, and then the term human betaretrovirus (HBRV) was adopted when a similar agent was characterized in patients with primary biliary cholangitis (PBC), an autoimmune liver disease. In mice, MMTV is an established cause of breast cancer, lymphoma, and renal cancer and is linked with several inflammatory disorders in genetically manipulated or outbred models [1,2]. In contrast, HBRV is not recognized as a “cause” of either breast cancer or PBC by the scientific community. The main challenge in linking HBRV with human disease is a lack of reproducible diagnostic assays to confirm infection; an issue now being addressed. In addition, the low viral burden and similarity of human and mouse isolates have prompted concern about mouse DNA contamination in human PCR studies. Additionally, the poor humoral responses elicited by infection have minimized the utility of diagnostic ELISA assays. Nevertheless, the demonstration of proviral integrations in human samples and the isolation of a transmissible betaretrovirus from patient samples provide sufficient support for HBRV as an infectious agent in humans [1,2]. Therefore, this Special Issue provides an opportunity to share new data and help bridge the gap in describing the potential role of HBRV in human disease.

The history of HBRV was broadly outlined by all contributors, with some expressing the view that it is not possible to prove a causal association in complex disorders with multiple etiological influences. Lawson and James reviewed the relationship between HBRV and breast cancer using the structured Hill’s criteria to build a plausible relationship between infection and the disease [3]. The same approach was taken to link HBRV with the autoimmune liver disease, PBC [2]. Parisi and colleagues provided a synopsis of the MMTV life cycle, resistance to infection, and the viral pathogenesis of breast cancer and lymphoma [4]. Generoso Bevilacqua presented an encyclopedic overview of the history of MMTV, breast cancer in mice, and then the development of clinical diseases and background epidemiology related to betaretrovirus infection in humans [5].

To date, HBRV is the only exogenous betaretrovirus characterized in humans. Goubran and colleagues isolated HBRV from PBC patient lymph nodes and then transmitted the virus to cholangiocytes, the target of the immune-mediated disease process for patients with PBC [1]. Syed and colleagues reviewed the detection of “gold standard” evidence of retrovirus infection with the identification of over 2000 HBRV proviral integrations in liver disease patients’ samples and re-evaluated the potential role of HBRV in triggering autoimmunity to mitochondrial enzymes, which is in part related to metabolic remodeling [2].

Much is still to learn about HBRV, including the epidemiology and the route of transmission. Stewart and Chen retraced their steps to update the zoonosis hypothesis by relating mouse population outbreaks with a spike in breast cancer prevalence [6]. As MMTV and HBRV are genetically indistinguishable, it is currently not possible to determine the provenance of specific isolates (see commentary by Gunzburg and Salmons) [7]; however, HBRV Envelop sequences from PBC patients appear to maintain amino acid sequences only found in human isolates [1]. Parisi and colleagues provided an interesting perspective in that zoonosis may occur through pets [4], whereas Bevilacqua reiterated the potential of HBRV horizontally spreading to humans, citing the high prevalence of HBRV in saliva and the detection of HBRV in the dental callus of skulls dating back to the copper age [5].

With a focus on future treatment, Hochman and Braitbard evaluated the oncogenic role of MMTV envelope precursor protein (p14) in lymphoma and breast cancer [8]. They reviewed the use of passive immunization and active vaccination to target p14-expressing cancer, thus providing the potential for translational management of similarly antigenic human cancers. Aiming at intervention, HIV antiretroviral therapy has been repurposed to treat murine cholangitis related to MMTV and provide clinically meaningful responses in PBC patients with HBRV [2,9].

As Guest Editor, I am delighted to present this impressive collection of articles describing “Human Betaretrovirus and Related Diseases”. Notably, breast cancer and PBC share several commonalities; both have a female predominance, shared genetic risk alleles, a relationship with female hormones, and an increasing worldwide prevalence. Indeed, current estimates report a predicted rise in breast cancer burden by 40%, with more than 3 million new cases per year by 2040. This will lead to a 50% increase in deaths, exceeding 1 million per year—for a disease that may be preventable by vaccination [8]. These data underscore the importance of further study of the linkage of HBRV with human disease, as there is much to learn about the viral biology, pathogenesis, and epidemiology. I am confident that the collected articles will inspire more research—including the study of the fundamental biology of HBRV in oncogenesis and autoimmunity, as well as further viral prevalence studies with newly developed immune assays [2].
